# Exploring socio-demographic, physical, psychological, and quality of life-related factors related with fear of cancer recurrence in stomach cancer survivors: a cross-sectional study

**DOI:** 10.1186/s12885-022-09507-2

**Published:** 2022-04-15

**Authors:** Jinyoung Shin, Dong Wook Shin, Jungkwon Lee, JiHye Hwang, Ji Eun Lee, BeLong Cho, Yun–Mi Song

**Affiliations:** 1 Department of Family Medicine, Research Institute On Healthy Aging, Konkuk University Medical Center, Konkuk University School of Medicine, Seoul, South Korea; 2 Department of Family Medicine, Samsung Medical Center, Sungkyunkwan University School of Medicine, 81, Irwon–ro, Gangnam–gu, 06351 Seoul, South Korea; 3 Bucheon Geriatric Medical Center, Bucheon, South Korea; 4 Department of Physical and Rehabilitation Medicine, Samsung Medical Center, Sungkyunkwan University School of Medicine, Seoul, South Korea; 5 Department of Family Medicine & Health Promotion Center, Seoul National University Hospital, Seoul, South Korea

**Keywords:** Stomach neoplasms, Survivor, Fear, Recurrence

## Abstract

**Background:**

Stomach cancer is one the most common neoplasms with high mortality. However, fear of cancer recurrence (FCR) in stomach cancer survivors has been scarcely evaluated. Thus, the aim of this study was to evaluate FCR and factors related to FCR in Korean stomach cancer survivors.

**Methods:**

A total of 363 stomach cancer survivors who had completed primary treatment and had no metastasis or recurrence were recruited between September 2014 and March 2017 regardless of time lapse after the initial diagnosis. FCR was assessed using the Korean version of the FCR Inventory (FCRI). Participants were divided into two groups; clinical FCRI group (score of severity subscale of FCRI ≥ 13) and non-clinical FCRI group (the scores < 13). Socio-demographic factors, cancer stage, treatment, psychological factors, health-related quality of life (HRQoL), and health promotion and disease prevention behaviors were obtained using a self–administered questionnaire supplemented with face-to-face interview to fill out incomplete information. Factors associated with FCR were evaluated using linear regression analysis and multiple logistic regression analysis after adjusting for age, sex, cancer stage, time since cancer diagnosis, family cancer diagnosis, and comorbidities.

**Results:**

Average (standard deviation) time interval between cancer diagnosis and study participation was 7.3 (3.2) years. The distribution of socio-demographic and cancer–related factors did not differ according to the level of FCR. The higher FCRI level was associated with lower levels of social support (β: -0.190, *p* < 0.001), lower emotional function (β: -0.356, *p* < 0.001), more severe fatigue (β: 0.333, *p* < 0.001), more sleep problems (β: 0.299, *p* = 0.002), higher anxiety (β: 0.443, *p* < 0.001), and higher depression (β: 0.207, *p* < 0.001). However, clinical level of FCR was not associated with health promotion and disease prevention behaviors.

**Conclusions:**

FCR in stomach cancer survivors was associated with social, psychological, and HRQoL factors rather than demographic, socioeconomic, or cancer–related factors. This finding suggests that careful attention to FCR is necessary to provide more comprehensive survivorship care for stomach cancer survivors.

## Background

Stomach cancer is the 5^th^ most common neoplasm accounting for 5.6% of all new cancer cases. The number of deaths from stomach cancer is the 3^rd^ highest with 768,793 deaths according to GLOBOCAN 2020 data [1]. Stomach cancer is the most prevalent cancer in Korean males aged 35–64 years, with an estimated 27,005 new cases in 2020 [2]. Meanwhile, the five–year survival rate for stomach cancer was surprisingly increased from 43.8% in 1993–1995 to 77.0% in 2014–2018 [3]. The survival rate for stomach cancer has improved probably due to the active disease prevention strategy of the Korean National Health Insurance Service which provides free biennial health examinations for early detection of stomach cancer to all Korean citizens aged ≥ 40 years. Advanced treatment technology based on accumulated clinical experiences has also contributed to the improved survival rate of stomach cancer patients. Such improvement in survival rate of stomach cancer patients does not mean that we could overlook stomach cancer survivors’ problems because stomach cancer survivors are very likely to suffer chronically from various physical and psychosocial problems such as anemia, bone disease, weight loss, and emotional distress [4]. Especially, fear of cancer recurrence (FCR) is one of the most prevalent unmet psychosocial needs related to psychological distress, functional impairments, and increased use of health care resources [5, 6]. Health-related quality of life (HRQoL) can be defined as patients’ subjective perception of the impact of their diseases and their treatment in daily life including physical, psychological, and social functioning and well-being. It is closely related to the FCR of cancer survivors [7, 8]. Therefore, assessing FCR and identifying relevant factors are essential for cancer survivors’ care [9]. Higher FCR is associated with younger age, higher education status, uncontrolled physical symptoms, and psychological distress [10]. However, findings about associations of FCR with sex and marital status are inconsistent [6]. Furthermore, factors associated with FCR of cancer survivors may differ by cancer types [6, 8].

Stomach cancer is significantly influenced by diet, behavioral, and lifestyle risk factors [11, 12]. Several modifiable behavioral factors such as smoking, alcohol consumption, physical activity, and dietary factors as well as communication and care coordination during the treatment period are associated with FCR in breast, prostate, colorectal, skin, and non-Hodgkin lymphoma cancer survivors [8, 13, 14]. Although stomach cancer itself is significantly influenced by diet, behavioral, and lifestyle risk factors, dietary and behavioral factors associated with FCR of stomach cancer survivors are not well known. Recently, it has been reported that communication and care coordination during the treatment period are associated with FCR [15].

Therefore, the objective of this cross-sectional study was to evaluate the association of FCR of stomach cancer survivors with various factors including socio-demographic characteristics, cancer–related information, physical symptoms, psychological distress, social support, and quality of life in Korean stomach cancer survivors. Effects of FCR on health promotion and disease prevention behaviors of Korean stomach cancer survivors were also evaluated.

## Methods

### Study participants

Study participants were recruited from a cohort study of Korean adult (≥ 19 years) cancer survivors to evaluate long-term health problems of cancer survivors from September 2014 to March 2017. Originally, the cohort study of Korean cancer survivors recruited 2,037 cancer survivors who had completed primary cancer treatment without metastasis or recurrence regardless of the time lapse after the initial cancer diagnosis or cancer type in two university affiliated hospitals. These two hospitals have separate cancer centers involved in cancer care of around 20% of Korean cancer patients. Most participants of the cohort study visited a cancer survivorship clinic due to their unmet health concerns or post-treatment surveillance after five years from their initial cancer diagnosis.

Among the enrolled 373 stomach cancer survivors who provided responses to all 42 items of the Korean version of Fear of Cancer Recurrence Inventory (FCRI), four survivors who were found to have distant metastasis at diagnosis and six survivors who had an endoscopic mucosal resection were excluded considering the difference of cancer stage and treatment modality. Thus, 363 stomach cancer survivors were finally included in this study.

### Study variables

Data on FCR, demographic, socioeconomic, and clinical characteristics, satisfaction with communication within the medical team and care coordination were obtained using a self–administered questionnaire. A trained research assistant helped some study participants to fill in some incompletely answered questions through a face-to-face interview if necessary. Data on FCR were obtained using a Korean version of FCRI [9]. FCRI is a multidimensional questionnaire composed of 42 items with seven subscale components of FCR. The total score of FCRI ranges from 0 to 168 [5]. These seven subscales reflect potential stimuli activating FCR (triggers), presence and severity of intrusive thoughts associated with FCR (severity), emotional disturbance associated with FCR (psychological distress), impact of FCR on important areas of functioning (functional impairments), self–criticism toward FCR intensity (insight), reassurance seeking such as thorough self–examination or repeated medical consultations (reassurance), and other strategies to cope with FCR (coping strategies). Among these seven subscales, severity subscale was developed as a FCRI short form by Simard and Savard in 2015 [16]. For the original version of FCRI, Cronbach’s alpha values of the seven subscales were: trigger, α = 0.90; severity, α = 0.89, psychological distress, α = 0.86; functioning impairments, α = 0.91; insight, α = 0.80; reassurance, α = 0.75; and coping strategies, α = 0.89 [5]. Cronbach's alpha coefficient for the FCRI–Korean version was 0.85 for the total scale and 0.77–0.87 for subscales [9].

Socio-demographic characteristics, including age at survey, age at cancer diagnosis, sex, monthly household income level (≥ 4,000,000 won per month, 2,000,000–3,999,999 won per month, and < 2,000,000 won per month), achieved education level (≤ 9 years, 10–12 years, and ≥ 13 years), marital status (married/with a partner and unmarried/without a partner), religion (yes or no), and Charlson’s comorbidity index calculated using 18 conditions excluding age, were obtained using a self–administered questionnaire [17]. Their medical records were reviewed for cancer–related information, such as time since cancer diagnosis, cancer stage, additional treatment modality (chemotherapy, and/or radiotherapy), and previous cancer diagnosis of family members (spouse, 1^st^ degree relatives, or none).

We assessed their satisfaction with the level of communication on harmony (1–5 points), interaction (1–5 points), and role responsibility (1–5 points) within the medical team, with a higher score indicating a higher level of dissatisfaction with communication within the medical team. Care coordination assessment was based on the following question: “Did you receive all care services that were necessary for dealing with your health concern during the cancer treatment period?” [15]. Scoring ranged from one to five, with a higher score indicating a higher level of dissatisfaction with care coordination. Duke-UNC functional social support questionnaire (FSSQ) was used to measure patient’s perceived need for a social support network [18]. It was composed of eight items for two subscales (confidant and effective support) with five answer choices (scores from five = ‘As much as I would like’ to one = ‘Much less than I would like’) [19]. The average score for eight responses was presented. The Cronbach’s α of the Korean version of the FSSQ was 0.89 [20].

The EuroQoL–visual analog scale (EQ-VAS) and the European Organization for Research and Treatment of Cancer Quality of Life Questionnaire version 3.0 (EORTC QLQ–C30) were used to assess HRQoL. The EQ-VAS is a standard vertical 20 cm visual analog scale for rating current HRQoL, with scores ranging from zero (worst imaginable) to 100 (best imaginable). EORTC QLQ–C30 is a 30–item questionnaire developed to assess HRQoL of cancer patients incorporating five functional scales (physical, role, emotional, cognitive, and social), a nine–symptom scale (fatigue, nausea and vomiting, pain, dyspnea, insomnia, appetite loss, constipation, diarrhea, and financial difficulties), and a global quality of life scale. Participants responded using a four-point or a seven-point Likert scale. The score of each scale ranged from zero to 100. Except for the cognitive functioning subscale, Cronbach's alpha coefficients of the Korean version of the EORTC QLQ–C30 subscale were greater than 0.70 [21]. Fatigue severity scale (FSS) was developed to assess fatigue in patients with chronic diseases using 10 questions rated from one (strongly disagree) to seven (strongly agree) [22], with a higher score denoting a more severe fatigue. The Cronbach’s α for total FSS was 0.935, ranging from 0.925 to 0.932 for its subscales [23]. Sleep problems were surveyed with a self-administered questionnaire. Levels of sleep problems were assessed based on mean frequency: 1) no problem, 2) problems 1–2 nights per week, 3) problems 3–4 nights per week, and 4) problems every night. Anxiety and depressive mood were evaluated with the Hospital Anxiety and Depression Scale (HADS) with scores ranging from 0 to 21. The Cronbach’s α of the Korean version of HADS was 0.89 for the anxiety subscale and 0.86 for the depression subscale [24].

To evaluate health promotion and disease prevention behavior following the recommendation for early detection and treatment of cancer by United States Preventive Service Taskforce and Korean Medical Association for stomach cancer survivorship care [25–27], we collected data on receipt of the second primary cancer screening test (colonoscopy, mammography, and Pap smear; yes/no), patterns for smoking, drinking, and exercise, bone mineral density evaluation within two years, supplementary drug intake, and dietary pattern changes after cancer diagnosis with a self-administered questionnaire. Quitting of smoking and drinking was evaluated for occurrence after a cancer diagnosis or treatment (yes/no). Regular exercise was defined as at least one time per week for 30 min. Supplementary drug intake was defined as consistently (more than 2 weeks) taking supplements such as vitamin B complex, vitamin C, vitamin D, multivitamins, calcium, and iron after a cancer diagnosis. Dietary pattern changes after cancer diagnosis were evaluated by asking whether the frequency of intake was increased or decreased for organic food, fruit or vegetables, pork or beef, processed meat, and salty food.

### Statistical analysis

Descriptive statistics of study participants are presented as mean value ± standard deviation for continuous variables and number (percentage) for categorical variables. A cut-off point for the FCRI Korean version to screen clinical levels of FCR has not been determined yet. Thus, we applied a score ≥ 13 for severity subscale (short-form FCRI) as a cut-off score to screen cancer survivors with clinical levels of FCR for the present study suggested by Simard et al., who developed FCRI [16]. After categorizing study participants into two groups based on the cut-off score (with clinical levels of FCR or without clinical levels of FCR), we compared the distribution of socio-demographic and clinical characteristics between the two groups by t-test for continuous variables and Chi-square test for categorical variables. We then evaluated the association of clinical levels of FCR with satisfaction with communication and care coordination, functional social support, HRQoL, fatigue severity scale, sleep problems, anxiety, and depression using linear regression analysis after adjusting for age, sex, cancer-related factors (family history of cancer, cancer stage, and time lapse since cancer diagnosis), and comorbidity. We further evaluated the association of clinical levels of FCR with health promotion and disease prevention behaviors using a multiple logistic regression analysis after adjusting for same covariates. These covariates were selected for regression models because they were suggested as confounding factors in a previous study [8]. In regression models, quality of life measures, social and psychological variables, and health promotion/disease prevention behaviors were inputted as dependent variables with clinical level of FCR as an independent variable. Since conducting multiple analyses on the same dependent variable, we set the significant level of *p*-value after Bonferroni’s adjustment to avoid an increased chance of committing a Type I error. All statistical analyses were performed using IBM SPSS Statistics for Windows, version 24.0 (IBM Corp., Armonk, NY, USA).

## Results

The mean age of subjects was 56.9 years old, with an average duration of 7.3 years since diagnosis. About 39.1% of study participants were classified into the clinical FCRI group. Table 1 shows FCRI scores, distribution of sociodemographic, and clinical characteristics of study participants according to the level of FCRI. Total and subscale scores of FCRI were significantly higher in the clinical FCRI group except for the scale of coping strategies. There were no significant differences in age, sex, income, education, marital status, religion, comorbidity, time since cancer diagnosis, cancer stage, treatment modality, or family history of cancer according to the level of FCRI.

**Table 1:** Distribution of sociodemographic and clinical characteristics according to the level of Fear of Cancer Recurrence Inventory in Korean stomach cancer survivors

Variables	Total	Non- Clinical FCRI	Clinical FCRI^a^	*P*-value^†^
Number of subjects	363	221(60.9)	142(39.1)	
FCRI score				
Total score (range: 0 – 168)	58.3 ± 24.3	45.5 ± 17.2	78.1 ± 20.0	< 0.001
Subscale score				
Triggers (range: 0 – 32)	13.1 ± 7.1	9.8 ± 5.7	18.2 ± 6.0	< 0.001
Severity (range: 0 – 36)	11.5 ± 7.2	6.7 ± 3.6	18.9 ± 4.6	< 0.001
Psychological distress (range: 0 – 16)	4.2 ± 4.0	2.4 ± 2.9	7.1 ± 3.8	< 0.001
Functioning impairments (range: 0 – 24)	4.8 ± 5.4	3.4 ± 4.6	7.0 ± 5.7	< 0.001
Insight (range: 0 – 12)	1.3 ± 2.1	0.5 ± 1.0	2.7 ± 2.5	< 0.001
Reassurance (range: 0 – 12)	4.8 ± 3.3	4.4 ± 3.4	5.4 ± 3.1	0.008
Coping strategies (range: 0 – 36)	18.6 ± 7.6	18.4 ± 8.1	18.9 ± 6.9	0.508
Age at survey, years	56.9 ± 9.6	58.1 ± 9.3	55.1 ± 9.9	0.252
Age at cancer diagnosis, years	49.6 ± 9.7	50.7 ± 9.4	47.7 ± 10.1	0.250
Sex				0.067
Male	193(53.2)	126(65.3)	67(34.7)	
Female	170(46.8)	95(55.9)	75(44.1)	
Household income (won/month)				0.344
≥ 4,000,000	167 (46.0)	94(56.3)	73(43.7)	
2,000,000–3,999,999	108 (29.8)	72(66.7)	36(33.3)	
< 2,000,000	88 (24.2)	55(62.5)	33(37.5)	
Achieved education level				0.429
0–9 years	65 (17.9)	41(63.1)	24(36.9)	
10–12 years	141 (38.8)	91(64.5)	50(35.5)	
≥ 13 years	157 (43.3)	89(56.7)	68(43.3)	
Marital status				0.488
Married/with partner	317 (87.3)	190(59.9)	127(40.1)	
Unmarried/without partner	46 (12.7)	31(67.4)	15(32.6)	
Religion				0.740
Yes	247 (68.0)	151(61.1)	96(38.9)	
No	116 (32.0)	70(60.3)	46(39.7)	
Carlson Comorbidity Index^b^				0.521
0	315 (86.8)	193(61.1)	123(38.9)	
1	35 (9.6)	19(57.6)	14(42..4)	
≥ 2	13 (3.6)	9(64.3)	5(35.7)	
Time since cancer diagnosis, years	7.3 ± 3.2	7.4 ± 3.1	7.3 ± 3.3	0.817
Cancer stage				0.386
I	242 (66.7)	146(60.3)	96(39.7)	
II	67 (18.5)	45(67.2)	22(32.8)	
III	54 (14.9)	30(55.6)	24(44.4)	
Treatment modality				0.367
Only surgery	224 (61.7)	137(61.2)	87(38.8)	
Surgery + Chemotherapy	60 (16.5)	34(56.7)	26(43.3)	
Surgery + Chemotherapy + Radiotherapy	79 (21.8)	50(63.3)	29(36.7)	
Cancer diagnosis of family member				0.395
Spouse	14 (3.9)	7(0.5)	7(0.5)	
1^st^ degree relatives	153 (42.1)	90(58.8)	63(41.2)	
None	189 (52.1)	120(63.5)	69(36.5)	

Data were presented as mean value ± standard deviation or number (row percentage)

^a^The score of FCRI short form (FCRI severity subscale) was ≥ 13

^†^*P* values were obtained by t-test or chi-square test

^b^The Carlson Comorbidity Index was calculated after excluding age and cancer

Table 2 shows associations of clinical level of FCR with communication, care coordination, functional social support, HRQoL, fatigue, sleep problem, and psychological distress, when age, sex, cancer stage, Carlson’s Comorbidity Index, and time since cancer diagnosis were adjusted. FCRI was inversely associated with social support (mean FSSQ, β: -0.190, *p* < 0.001), functional scales of EORTC QLQ–C30 (*p* ≤ 0.001) except role functioning scale. FCRI was positively associated with symptom scales of EORTC QLQ–C30, such as fatigue (β: 0.243, *p* < 0.001), pain (β: 0.183, *p* = 0.001), and financial difficulty (β: 0.167, *p* = 0.002), fatigue severity scale (β: 0.333, *p* < 0.001), sleep problems (β: 0.299, *p* = 0.002), anxiety (β: 0.443, *p* < 0.001), and depression (β: 0.207, *p* < 0.001).

**Table 2:** Association of the level of Fear of Cancer Recurrence Inventory with health-related quality of life and psychosocial factors in Korean stomach cancer survivors

		Overall (*N* = 363)	Non-clinical FCRI (*N* = 221)	Clinical FCRI^a^ (*N* = 142)	Association	
Measurement tools	range	Mean ± SD	Mean ± SD	Mean ± SD	Beta coefficients^b^	*P*-value
Communication unsatisfaction^d^	3–15	4.3 ± 1.8	4.1 ± 1.8	4.6 ± 1.8	0.092	0.057
Care coordination^d^	1–5	1.7 ± 0.9	1.6 ± 0.8	1.9 ± 0.9	0.080	0.295
Functional social support, mean^c^	1–8	3.0 ± 1.0	3.2 ± 1.7	2.5 ± 1.5	-0.190	** < 0.001**
Confidant support, total	5–25	15.0 ± 3.9	16.0 ± 3.6	13.4 ± 3.8	-0.164	**0.002**
Affective support, total	3–15	11.2 ± 2.9	12.3 ± 2.5	9.8 ± 2.7	-0.201	** < 0.001**
EQ_VAS^c^	0–100	68.9 ± 16.4	70.8 ± 15.7	66.6 ± 16.6	-0.126	0.019
EORTC QLQ-C30						
Global health status/QOL^c^	0–100	66.1 ± 17.3	67.9 ± 16.7	63.6 ± 17.4	-0.159	0.003
Functional scales^c^						
Physical functioning	0–100	81.7 ± 15.3	84.1 ± 14.4	78.0 ± 15.2	-0.175	**0.001**
Role functioning	0–100	86.9 ± 18.7	89.5 ± 16.7	83.2 ± 21.0	-0.154	0.004
Emotional functioning	0–100	79.4 ± 18.4	84.5 ± 15.7	70.9 ± 19.2	-0.356	** < 0.001**
Cognitive functioning	0–100	76.8 ± 17.8	79.4 ± 16.4	72.1 ± 18.3	-0.204	** < 0.001**
Social functioning	0–100	80.2 ± 22.8	84.7 ± 18.4	72.7 ± 26.1	-0.272	** < 0.001**
Symptom scales^d^						
Fatigue	0–100	34.9 ± 23.4	29.9 ± 21.9	43.0 ± 23.4	0.243	** < 0.001**
Nausea and vomiting	0–100	12.8 ± 17.0	10.6 ± 17.1	16.8 ± 17.8	0.150	0.004
Pain	0–100	14.2 ± 18.7	11.4 ± 17.9	18.6 ± 19.4	0.183	**0.001**
Dyspnea	0–100	16.0 ± 21.7	13.0 ± 19.7	20.4 ± 23.1	0.154	0.004
Insomnia	0–100	27.3 ± 31.6	23.9 ± 31.4	32.2 ± 30.6	0.135	0.011
Appetite loss	0–100	13.9 ± 21.5	11.9 ± 21.2	17.1 ± 22.4	0.107	0.047
Constipation	0–100	17.5 ± 23.1	16.3 ± 22.2	19.7 ± 24.8	0.060	0.264
Diarrhea	0–100	31.8 ± 26.4	28.7 ± 25.1	36.9 ± 27.7	0.121	0.020
Financial difficulties	0–100	18.4 ± 24.7	15.5 ± 22.4	23.0 ± 27.6	0.167	**0.002**
Fatigue severity scale^d^	1–7	2.8 ± 1.7	2.4 ± 1.5	3.4 ± 1.8	0.333	** < 0.001**
Sleep problems^d^	1–4	2.0 ± 1.1	1.9 ± 1.1	2.1 ± 1.1	0.299	**0.002**
Anxiety^d^	0–18	4.7 ± 3.3	3.5 ± 2.5	6.5 ± 3.5	0.443	** < 0.001**
Depression^d^	0–18	8.0 ± 3.6	7.4 ± 3.5	8.9 ± 3.6	0.207	** < 0.001**

Data were presented as mean value ± standard deviation

^a^The score of FCRI short form (FCRI severity subscale) was ≥ 13

^b^Estimated using linear regression analysis after adjusting for age, sex, cancer stage, Carlson’s Comorbidity Index, and time since cancer diagnosis. In the analysis, each health-related quality of life and psychosocial factor was put as a dependent variable and the level of FCRI was put as an independent variable.

Significant correlation below Bonferroni cut-off (*p* < 0.002) are in bold

^c^Higher score means better health status

^d^Lower score means better health status

Table 3 shows associations of clinical level of FCR with health promotion and disease prevention behaviors after adjusting for age, sex, cancer stage, Charlson’s Comorbidity Index, and time since diagnosis. Clinical level of FCR showed no significant association with disease prevention behaviors such as receiving second primary cancer screening and bone mineral density measurement for osteoporosis evaluation (*p* > 0.05). Clinical level of FCR showed no association with health promotion behaviors either such as smoking, alcohol intake, physical exercise, supplementary medicine use, or dietary pattern changes (*p* > 0.05).

**Table 3:** Association^a^ of the level of Fear of Cancer Recurrence Inventory with health promotion and disease prevention behaviors in Korean stomach cancer survivors

Health promotion and disease prevention behaviors	Odd ratio (95% confidence intervals)		*P*-value
	Non-clinical FCRI	Clinical FCRI	
Secondary primary cancer screening			
Colonoscopy	1	1.437(0.765,2.699)	0.259
Mammography (female)	1	1.109(0.515,2.386)	0.792
Pap smear (female)	1	0.682(0.320,1.455)	0.322
Bone mineral density measurement			
Total subjects	1	0.904(0.409,1.997)	0.803
Postmenopausal women and elderly men (≥ 70 years)	1	0.994(0.425,2.325)	0.990
Quitting smoking after cancer diagnosis	1	1.892(0.210,5.763)	0.262
Quitting drinking after cancer diagnosis	1	1.230(0.783,1.933)	0.369
Regular exercise (≥ once/week and ≥ 30 min/day)	1	1.727(0.764,3.906)	0.190
Supplementary drug intake (≥ 2 weeks after cancer diagnosis)			
Vitamins	1	1.077(0.658,1.764)	0.769
Calcium	1	0.443(0.110,1.776)	0.250
Iron	1	2.031(0.552,7.482)	0.287
Dietary pattern changes after cancer diagnosis			
Increased organic food intake	1	0.641(0.335,1.224)	0.178
Increased fruit intake	1	1.429(0.904,2.259)	0.127
Increased vegetables intake	1	1.274(0.810,2.003)	0.294
Reduced pork or beef intake	1	1.077(0.682,1.701)	0.750
Reduced processed meat intake	1	0.786(0.419,1.475)	0.454
Reduced salty food intake	1	1.251(0.752,2.079)	0.388

^a^Odds ratios and 95% confidence intervals were estimated using multiple logistic regression analysis after adjusting for age, sex, cancer stage, Carlson’s Comorbidity Index, and time since diagnosis. In the analytic model, each health promotion and disease prevention behavior was put as a dependent variable and level of FCRI was put as an independent variable

## Discussion

In this study, higher level of FCR in Korean stomach cancer survivors was associated with poor social support, functional decline, fatigue, pain, sleep problems, anxiety, and depression. However, FCR showed no significant relationship with cancer–related factors or preventive behaviors of cancer survivors.

As found in other cancer patients, uncontrolled physical symptoms, psychological distress, and low HRQoL of stomach cancer survivors were associated with clinical FCRI [8]. In a previous study with 342 Chinese breast cancer survivors who were diagnosed with cancer for less than 2 years (67.3% of the total number of subjects), between 2 and 3 years (14.0%), and more than 3 years (18.7%), cancer-related and socio-demographic characteristics such as no religious beliefs, lower family income, and treatment modality (breast-conserving surgery) were associated with higher levels of FCR [28], different from our study findings. Such difference might be due to the different distribution of time lapse after cancer diagnosis of study subjects between the Chinese study (less than 2 years in 67.3% of study subjects) and our study (average time interval between cancer diagnosis and study participation: 7.3 years), given the inverse association between FCR and time since diagnosis reported in a systematic review [8]. In addition, type of cancer may influence the association of FCR with cancer-related and socio-demographic characteristics. Breast cancer patients might have higher levels of FCR because they are frequently affected by psychological and social factors [29]. In our study, levels of FCR in stomach cancer survivors were found to have significant associations with social supports and social or emotional quality of life. Considering that social support might be related to mood, coping strategies, and positive adjustment in cancer survivors [30–32], the association between social support and FCR seems very plausible. However, interpretation for the social support – FCR association has to be very cautious because various findings could be observed for the association across different study populations due to cultural differences such as perceived support level and role of family [32, 33].

We found that FCR did not differ by sex in this study. A systematic review including 43 studies has reported similar findings to our study, although the authors of that systematic review have argued that further research is needed because gender-specific researches included in the review article were insufficient [6]. On the contrary, in a Canadian study of patients with breast, prostate, lung, and colorectal cancers, FCR levels of female cancer survivors were higher than those of male survivors [5]. Recent studies of hematologic cancer patients including 467 Korean lymphoma patients have also reported that female patients had a greater FCR than male patients [33, 34]. We think that the null association between sex and FCR in our study might have been observed because known prognosis of a cancer could influence emotion [35]. Stomach cancer is one of the cancers with a good prognosis in Korea. Around 85% of our study participants were diagnosed with stage 1 or 2 stomach cancer. In this case, the five-year survival rate can be expected to be up to 97.4% in males and 98.8% in females. On the other hand, the five-year survival rate of Korean non–Hodgkin lymphoma patients is much worse [3]. Therefore, FCR difference between males and females might be indistinct in survivors of a cancer showing good prognosis, such as early stomach cancer [33, 35].

Regarding the relationship between education and FCR, findings were inconsistent. Higher FCR was associated with a lower education level in a study of American breast cancer patients [36]. It has been suggested that highly educated patients might have a greater understanding of cancer diagnosis with more effective coping strategies [6, 37]. However, a systematic review of studies on adult cancer survivors found no association between education level and FCR [8], similar to our study results. Therefore, further studies are needed to evaluate the relation between FCR and education level.

Several studies have found no association of FCR with treatment type, duration, or disease stage of cancer [8, 33]. However, physical and psychological symptoms such as fatigue, pain, sleep problems, distress, anxiety, and depression were strongly associated with FCR regardless of cancer type or time since cancer diagnosis [8]. FCR of long-term cancer survivors could be affected by uncontrolled physical and psychological symptoms [8, 9, 29]. Therefore, continuous concern for controlling their physical and psychological problems seems important in aspects of both FCR management and care coordination [15].

Well-coordinated care may reflect a good relationship between patients and healthcare providers. Although we could not find a significant association between care coordination and FCR, a provision of proper coaching or strategies for overcoming FCR by healthcare providers were reported to lower the level of FCR [15]. Additionally, whole-person care and tailored patient education for lifestyle modification might reduce the development of comorbidities and prevent late adverse effects after cancer diagnosis [38]. The association between FCR and care coordination was observed in a study of 2,290 non–metastatic multi-ethnic breast cancer patients [39].

Interestingly, we observed that health promotion and disease prevention behaviors such as lifestyle modifications, secondary primary cancer screenings, and surveillance of comorbidities did not affect FCR in stomach cancer survivors with clinical FCRI. Our findings do not support the ‘teachable moment theory,’ which suggests that cancer survivors who experience greater FCR are motivated to engage in healthy behaviors [14, 40]. After diagnosis of cancer, survivors have a chance to learn how to look back on their wrong habits and likely to initiate healthy diet, exercise, smoking cessation, and weight control to improve overall health [41]. However, FCR severity might neither encourage nor hinder health behaviors of cancer survivors [14]. For example, a study in the United States found that survivors of breast, gynecologic, colorectal, and non-melanoma skin cancers with high FCR were twice more likely to smoke and less likely to do enough physical exercise than those with lower FCR [13]. Several reasons could be hypothesized for these findings. First, cancer survivors with high FCR might experience high levels of distress. Thus, they may not adhere to healthy lifestyle recommendations [42]. Second, most cancer survivors try to improve their health behaviors regardless of the level of FCR. The practice of healthy habits was found to have doubled after cancer diagnosis regardless of the level of FCR in US survivors [13]. The rate of unhealthy behaviors in our study was too low to determine significant differences across different FCR groups. The proportion of current smokers in our study was lower (0.4%) than that (14%) in the United States study [13]. Last, the effect of FCR on health behaviors might be motivational for a certain period such as only during a treatment period. FCR is known to stabilize over time [8]. A study in the United Kingdom that tracked behaviors of cancer survivors over time in cancer survivors and controls revealed no group difference in lifestyle behaviors such as alcohol use, smoking, or physical activity, suggesting that FCR might not significantly affect health behaviors of long-term survivors [43]. Unfortunately, we did not find a variance in FCR according to time since diagnosis in this study.

The present study has some limitations. First, our study results might not be generalizable to non-Koreans or all stomach cancer survivors because of the following reasons: this study was conducted in Korean academic hospital setting; proportion of survivors from advanced stage stomach cancer were relatively low; many cancer patients in South Korea receive a regular medical examination; duration since gastrectomy was relatively long in our study subjects. Therefore, subject characteristics should be taken into account when interpreting findings of our study. Second, this study was conducted using a cross-sectional study design with information collected simultaneously. We could not ensure time relationships of FCR with satisfaction with communication and care coordination, health promotion behaviors, or disease prevention behaviors. Third, we collected study data using two methods: self-administration of questionnaire and face-to-face interview. There might be potential biases from using face-to-face interviews for some participants who filled out incomplete questionnaires because social desirability was reported to be higher in face-to-face interviews [44]. The number of face-to-face interviews were not counted and that interviews were not administered to all participants, which could be a limitation. Fourth, we could not compare the effect of treatment modality (e.g., endoscopic mucosal resection versus surgical treatment) on FCR because a too small number (*n* = 6) of patients receiving endoscopic treatment was included in our study. In a previous study of early esophageal cancer survivors, endoscopic treatment exerted a less negative impact on the quality of life and physical symptoms than open surgery [45]. Considering the effect of treatment modality on FCR, further study including more stomach cancer survivors who have received endoscopic mucosal resection treatment would be needed. Lastly, clinical cut-off score adopted for the present study (severity subscale score ≥ 13) might have been inadequte to categorize clinical levels of FCRI in our study subjects. A previous study reported that FCRI-SF score was significantly higher in English language samples (18.0; 95% CI: 16.0–20.0) than in non-English language samples (14.3; 95% CI: 12.9–15.7; Beta = 3.7; *p* = 0.003), suggesting that clinical cut-off score could also differ across different populations [46]. Therefore, further study might be needed after setting a clinical cut–off point for FCRI in Korean stomach cancer survivors.

Nevertheless, this study is the first to evaluate factors related to FCR in stomach cancer survivors. It has the strength to consider a wide range of factors, including cancer-related and socio-demographic characteristics, physical symptoms, psychological distress, quality of life, and health promotion or disease prevention behaviors.

## Conclusions

FCR of stomach cancer survivors was associated with physical symptoms, psychological distress, social support, and HRQoL. Findings of this study can help us better understand the FCR of stomach cancer survivors and make a more comprehensive survivorship care plan.

## Data Availability

The datasets used and/or analyzed during the current study are available from the corresponding author on reasonable request.

## Abbreviations

FCR: Fear of cancer recurrence
FCRI: Fear of cancer recurrence inventory
HRQoL: Health-related quality of life
EQ-VAS: The EuroQoL–visual analog scale
EORTC QLQ–C30: The European Organization for Research and Treatment of Cancer Quality of Life Questionnaire version 3.0
FSS: Fatigue severity scale
HADS: Hospital Anxiety and Depression Scale
FSSQ: Functional social support questionnaire
